# Compliance of biosecurity practices for compartmentalization to foot-mouth disease and classical swine fever viruses in commercial swine companies from southern Brazil

**DOI:** 10.3389/fvets.2023.1125856

**Published:** 2023-03-08

**Authors:** Ana Paula Serafini Poeta Silva, Kori Khan, Luís Gustavo Corbellini, Antônio Augusto Medeiros, Gustavo S. Silva

**Affiliations:** ^1^Veterinary Diagnostic and Population Animal Medicine, College of Veterinary Medicine, Iowa State University, Ames, IA, United States; ^2^Department of Statistics, Iowa State University, Ames, IA, United States; ^3^Corb Science, Porto Alegre, Rio Grande do Sul, Brazil; ^4^Secretaria de Agricultura, Pecuária e Desenvolvimento Rural, Porto Alegre, Rio Grande do Sul, Brazil

**Keywords:** compartment, foreign animal disease, swine industry, cluster analyses (CA), spatial modeling

## Abstract

Classical swine fever (CSF) and foot-mouth disease (FMD) are both highly contagious disease and disruptive to commercial trades, but they are examples of foreign animal diseases that biosecurity-based compartmentalization could be used to support trade in free zones in response to an outbreak. This study aimed to evaluate biosecurity compliance to the Federal Normative Instruction #44 from December 4th, 2017 (BRAZIL, 2017) in commercial swine farms located in southern Brazil. A total of 604 swine farms from 10 commercial swine companies were sampled, from which 28.5% were breeding farms, 29.1% nursery, 32.8% finishing, 6.8% multipliers, and 2.8% farrow-to-finish. Cluster analyses revealed that farms with high compliance (*n* = 303, Cluster 1) performed 71% of the practices, moderate (*n* = 219, Cluster 2) 47%, and the low (*n* = 82, Cluster 3) 33%. A spatial logistic regression model estimated that biosecurity compliance was highest in only one of 10 commercial swine companies, and within a company, multipliers (when present) obtained the highest biosecurity compliance (*p*-value < 0.01). These results suggest that major improvements in biosecurity practices are needed in breeding herds, nursery, and grow-finish farms to be compliant to the Federal Instruction #44. Based on the combination of these analyses, only one commercial swine company was more suitable to establish compartments for CSF and FMD with minimal investments. Still, this study revealed that the majority of commercial swine companies needs to improve biosecurity practice protocols to then target compartmentalization.

## Introduction

Due to the continuous threat of foreign animal diseases in free zones, livestock companies within a country or region may implement compartmentalization strategies, as proposed in the OIE Terrestrial Code (1). The guidelines in the Terrestrial Animal Code by World Organization Animal Health (WOHA) states that compartment is an alternative to manage foreign animal disease in the population without discontinuing trade (2). Areas of compartmentalization are implemented by establishing a common biosecurity management system among a subpopulation with a distinct health status related to a specific disease(s) requiring surveillance for international trade (1). That is, compartmentalization is not limited to spatial locations of farms but also epidemiological aspects of disease control, e.g., consistent and well-executed biosecurity practices, surveillance through diagnostic testing, availability of breeding stock to continuously trade, and animal traceability. These aspects entail that compartment viability and continuity hinge on a robust relationship between industry and veterinarian authorities. Further, a precedent contingency plan defining specific actions to be taken if the foreign animal disease(s) is detected and procedures for restoring the compartment's health status is also an important step in this process (3).

The verticalization of swine and poultry industries is known to be a facilizing factor in creating compartments. Accordingly, the poultry industry includes several examples of overseen and ongoing compartments for H5/H7 Avian Influenza and New Castle Virus Disease in Brazil, the Netherlands, the U.S.A., and the U.K. (4, 5). Regarding swine industries, federal veterinarian authorities have started implementing plans and adequate internal legislation based on the WOHA's guidelines for compartmentalization for CSF and FMD. For instance, Brazil implemented the Federal Normative Instruction #44 in 2017 (6), while Argentina implemented Resolution 192/2021 (7), which allows and guides swine companies to compartmentalization in accordance with WOAH (2). Likewise, the Secure Pork Supply (SPS) plan is seen as an initial step and instrument for compartmentalization in the U.S.A. (8). For all these industries, a precise assessment of current infrastructure and biosecurity compliance in swine farms is still needed to promote compartmentalization (9).

Amass and Clark (10) described that biosecurity practices include procedures to preclude disease-causing agents from entering or spreading into a herd or barn. It is recognized that adopting these procedures will reduce economic losses given the occurrence of infections and protect territories nationally against foreign animal diseases (11). Amass et al. (12) reported that foot-mouth disease (FMD) transmission to susceptible pigs and sheep was prevented once farm personnel showered and wore clean clothing prior to contact. Some biosecurity practices are more critical or more often adopted in swine farms. Silva et al. (13) demonstrated that practices related to internal biosecurity were more frequently implemented. While farms including bio-exclusion biosecurity practices, such as “feed bin outside of the barn limit,” “perimetral fences around farm or barn(s),” and “transit of trucks inside the farm is prohibited,” obtained overall higher biosecurity scores. Further, in this same study, it was shown that general farm characteristics, such as population size, farm size, farm age, and education level of farm owner, as well as operation production type, and commercial swine company played a role in biosecurity compliance in swine farms in southern Brazil.

Brazil is one of the most important pork producers, e.g., the fourth-largest producer and exporter of pork meat globally, with 3,983 megatons of pork produced in 2019 (14). Approximately 90% of Brazilian pork production is concentrated in the south, southeast, and midwestern states (14) in which the WOHA recognizes them as a free zone from African Swine Fever (ASF), Classical swine fever (CSF), and FMD. However, Santos et al. (15) revealed that international borders in Rio Grande do Sul State (RS, in southern Brazil) are at risk of FMD entry. Furthermore, currently, northeastern states in Brazil have been undergoing CSF outbreaks (16), posing an ongoing threat to CSF free zones.

CSF and FMD are an economical burden and disruptive to international and regional commercial trades. CSF is caused by a *Pestivirus, Flaviviridae* family, which affects specifically members of the *Suidae* family (e.g., domestic and wild pigs) and is endemic in some countries or regions of Asia, Central and South America (17). While FMD is caused by an *Aphthovirus, Picornaviridae* family, and can affect cattle, swine, sheep, goats, and other cloven-hoofed ruminants. It is estimated that FMD virus is circulating in 77% of the global livestock population, e.g., countries and regions in Africa, the Middle East, and Asia (17). CSF and FMD are highly contagious transboundary diseases notifiable to WOAH. They are considered two of the most challenging diseases for livestock and wild animals because of their rapid spreading and tremendous impact on animal health. Thus, rapid and continuous efforts is vital to preclude CSF and FMD enter free regions, such as robust biosecurity programs comprised of quarantines, import requirements, movement restrictions (personnel and animals), cleaning and disinfection of equipment and vehicles, and vaccination.

Therefore, due to the potential threat of CSF and FMD introduction, companies can be prepared to rapid response to outbreaks by creating compartments to avoid disruption in the food supply chain. There is a need to understand the viability of its implementation in southern Brazil and investments that will need to be in place. The objectives of this study were: (1) to evaluate biosecurity compliance in swine farm as required by the Federal Normative Instruction (FNI) #44 (BRAZIL, 2017) (6), and (2) to investigate companies that would be suitable to create compartments.

## Materials and methods

### Study and sampling design

As described elsewhere (13), data used in this study was derived from a cross-sectional study to describe biosecurity practices and their relevance in commercial swine farms (*n* = 604) from RS in 2015. RS is located in the southern region and currently is the second largest pork exporter in Brazil (18), and the majority of commercial swine farms are integrated in companies. Accordingly, biosecurity practices are implemented to isolate commercial farms within a company from other companies, non-commercial (backyard farms), or independent swine producers. All procedures of this study were approved by the Scientific Research Committee from the Rio Grande do Sul University (#30594). Table 1 shows the number farms from the sample and source population by operational production type.

**Table 1:** Number of swine farms sampled based on the target population as described in Silva et al. (13).

	**Operation production type**					
	**Multipliers**	**Breeding**	**Nursery**	**Finishers**	**Farrow-to-finisher**	**Total**
Sample	41	172	176	198	17	604
Target population	41	381	269	3,235	17	3,943
Sample fraction	100.0%	45.9%	65.4%	6.1%	100.0%	7.7%

### Biosecurity practices survey and compartmentalization certification

A survey was applied *in locu* by trained veterinarians from the RS Official Veterinary Services (RS-OVS). The survey was comprised of an interview with farm owner or farm manager and an audition of biosecurity compliance, e.g., trained personal visually evaluated external facilities associated with biosecurity. Upon absence of the farm owner or farm manager (or whether both refused to participate), the auditor replaced the selected farm by the closest equivalent operation type pertaining to the equivalent company. The detailed description of biosecurity questionnaire can be found elsewhere (13). In brief, the questionnaire included 35 questions (yes or no) with the most relevant biosecurity practices performed in Brazil, U.S.A., and Europe, coupled with certification requirements for foreign animal diseases.

The FNI #44, from December 4th, 2017 (6) establishes standard procedures for compartment certification to CSF and FMD in Brazilian swine farms, e.g., following the WOHA's guidelines for compartmentalization (2), in which Chapters II and III define the minimum infrastructure and biosecurity practices required in swine farms, encompassing 21 items (Table 1).

### Statistical analyses

#### Cluster analysis

A *K*-means cluster analysis was performed to group farms based on their compliance to the biosecurity practices. Firstly, a binary variable for each of the 21 biosecurity practices of interest to the FNI #44 was created, e.g., “1” for compliance, 0, for non-compliance. Thereafter, the cluster analysis determined homogenous swine farm subgroups based on biosecurity compliance to CSF and FMD certification. A *K*-means clustering analysis was performed using binary matrix distance and Ward-D2 method considering all the 21 biosecurity practices described in Table 1. The analysis was performed using *NbClust* R package in R (19).

#### Spatial modeling

To evaluate the effect of swine company and operation production type on biosecurity compliance, a spatial logistic regression model was used. Because this model incorporated spatial dependence, only swine farms with spatial location information (*n* = 595) were included. To introduce the model, *Y*_*i*_denoted the number of biosecurity practices the *i*_th_ swine farm was compliant. The latitude and longitude of the *i*_th_ swine farm was defined as *s*_*i*_. The probability of the *i*_th_ swine farm being compliant with biosecurity practices to the swine company and operation production type was estimated using generalized additive model (20). More specifically, it was assumed that *Y*_*i*_~*B in* (21, *p*_*i*_) for *i* = 1, …, 595. It was denoted *A* to be a 595 × 14 model matrix, where the *i*th row contains the covariate information (swine commercial company and operation production type) for the *i*th swine farm. the model matrix assumed that swine Company 7 and breeding herd were the reference groups. The model was, as follows:


(1) $$\begin{array}{l} \text{logit}\left(\frac{p_{i}}{1-p_{i}}\right)=A_{i}\theta +f\left({\text{s}}_{i}\right), \end{array}$$

Where *A*_*i*_ was the row of the model matrix, corresponding to the *i*th swine farm, θ was the corresponding vector of unknown parameters, and *f*(*s*_*I*_) was an unknown, bounded function defined on an open bounded domain Ω ⊂ *R*^*d*^ which includes all locations *s*_1_, *s*_2_, …, *s*_595_. To arrive at estimates for $\hat{\theta }$ and $\hat{f}$ for θ and *f*, respectively, it was used thin plate splines with a basis size of 60. That is, the smoothness parameter λ was selected based on generalized cross-validation criterion (13).

Because all our covariates can be represented with dummy variables indicating the swine company and operation production type, the regression coefficients, θ had a straightforward interpretation. In this case, $\frac{\exp\left\{\theta_{k}\right\}\;}{1+\exp\left\{\theta_{k}\right\}\;}\;$ represented the probability for swine company or operation production type, after controlling for spatial dependence. The 95% confidence interval (CI) for probabilities were estimated by accounting the covariance matrix of spatial dependence.

Marginal probabilities were then used to compare differences between categories within a predictor. The interaction term between swine commercial company and operational production type was initially tested, but did not converge because eight of 10 companies did not have all types of operational production types, and the other two companies had as few as one observation for some operational production types. The analysis was carried out with the R package *mgcv* (21).

## Results

### Descriptive analyses of biosecurity practices

The 21 biosecurity practices for compartmentalization for CSF and FMD, as required by FNI #44 (BRAZIL, 2017) under WOAH's compartmentalization guidelines, are shown in Table 2. Overall, practices related to sanitation obtained higher frequencies of biosecurity compliance, e.g., 97.3% of all sampled farms included “Carcass disposal follows Official Veterinary Service's guidelines,” followed by 96.9% of all sampled farms included “Manure disposal following Official Veterinary Service's guidelines” and 96.2% perform periodically “Rodent control”.

**Table 2:** Biosecurity practices required in classical swine fever virus and Foot-month disease virus compartment certification, as described in chapters II e III from Federal Normative Instruction #44 (BRAZIL, 2017), and their frequency mean and standard error (SE) within the cluster.^a^

	**Total collected**	**Overall compliance (%)**	**High compliant**		**Moderate compliant**		**Low compliant**	
**Number of pig farms**	**604**		**303 (50.1%)**		**219 (36.3%)**		**82 (13.6%)**	
**Biosecurity practices**								
**I. Farm surroundings**			**%**	**SE**	**%**	**SE**	**%**	**SE**
Barn and/or farm is surrounded by fences	598	310 (51.8%)	84.4%	0.03	8.1%	0.02	27.5%	0.06
Farm has unique entrance	593	272 (45.9%)	78.9%	0.04	12.9%	0.03	21.9%	0.07
Do not raise other animal species with commercial purposes	604	173 (28.6%)	65.6%	0.04	68.5%	0.04	46.4%	0.07
Neighbors do not own or raise pigs	590	240 (40.7%)	67.7%	0.04	51.5%	0.05	38.5%	0.07
Staff personnel do not have contact with other livestock species	576	452 (78.5%)	27.1%	0.04	8.0%	0.02	9.9%	0.04
**II. Barriers**								
Disinfection chamber for materials	603	73 (12.1%)	10.4%	0.03	5.1%	0.02	9.5%	0.05
Disinfection ford for vehicles	595	37 (6.2%)	4.0%	0.01	0.0%	0.01	0.0%	0.01
Shower-in	598	278 (46.5%)	73.7%	0.04	12.4%	0.03	18.4%	0.06
Staff personnel are provided with farm-specific clothes and boots	601	247 (41.1%)	56.9%	0.04	20.0%	0.04	11.9%	0.05
Visitors are provided with farm-specific clothes and boots	587	250 (42.6%)	54.1%	0.04	13.6%	0.03	9.4%	0.04
Visitors are provided with guidelines prior entering the farm	582	260 (44.7%)	48.9%	0.04	32.3%	0.04	13.5%	0.05
Staff personnel are provided with periodic training in practices of pig management and health	592	455 (76.9%)	79.8%	0.04	78.0%	0.04	30.3%	0.07
**III. Sanitation**								
Periodic chemical water treatment (chlorine)	601	418 (69.6%)	88.9%	0.02	75.4%	0.04	50.0%	0.07
Periodic chemical-physical analyses of water provided to pigs	600	331 (55.2%)	59.3%	0.04	52.5%	0.04	25.7%	0.07
Carcass disposal follows Official Veterinary Service's guidelines	604	588 (97.3%)	99.8%	0.01	98.2%	0.01	92.9%	0.04
Manure disposal follows Official Veterinary Service's guidelines	604	585 (96.9%)	94.7%	0.02	98.1%	0.01	93.9%	0.04
Rodent control	599	576 (96.2%)	97.5%	0.02	98.4%	0.01	87.5%	0.05
Flies control	603	464 (76.9%)	71.2%	0.04	79.6%	0.04	34.8%	0.07
**IV. Trucks and feed**								
Trucks are dedicated to each production type	592	511 (86.3%)	90.6%	0.03	91.9%	0.03	40.4%	0.07
Transit of trucks inside farm area is prohibited	600	342 (57.0%)	86.7%	0.03	30.9%	0.04	32.8%	0.07
Feed formulation follows Official Veterinary Service's guidelines	600	600 (100%)	99.7%	0.01	99.9%	0.01	99.8%	0.01

a Percent value represent the frequency of a given biosecurity practice related to the total of swine farms within the cluster that included the biosecurity practice. For example, “High complaint” cluster included a total of 303 swine farm in which 84.4% farms were compliant to “Barn and/or farm is surrounded by fences.”

Custer 1 as “high compliant,” Cluster 2 as “moderate compliant,” and Cluster 3 as “low compliant.”

Related to farm surrounding practices, the two mostly performed biosecurity practices were “Staff personnel do not have contact with other livestock species” (78.5%) and “Barn and/or farm is surrounded by fences” (51.8%). For example, across companies, both practices were most frequently observed in farms from Company 5 (86.7% of farms included “Staff personnel does not have contact with other livestock species” and 73.3% of farms included fences), followed by Company 7 (61.5 and 73.1%). Across operation production types, 35.7 and 92.9% of all multipliers (*n* = 41), 62.2 and 50% of breeding herds (*n* = 172), 80.7 and 58.5% of nurseries (*n* = 176), 87.8 and 39.6% of finishers (*n* = 198), and 88.2 and 23.5% of farrow-to-finish farms (*n* = 17) included “Staff personnel does not have contact with other livestock species” and “Barn and/or farm is surrounded by fences”, respectively.

Related to barriers, the most compliant biosecurity was “Staff personnel are provided with periodic training in practices of pig management and health” (76.9%). Farms from Company 1 (*n* = 70) resulted in the highest frequency of periodic training for personnel (85.7% of farms), followed by Company 7 (80.8%) and 5 (80%). Across operation production types, 90.5% of all multipliers, 82.6% breeding herd, 75.0% nurseries, 68.5% finishers, and 47.1% farrow-to-finish farms included personnel training. In contrast, only 6.2% (*n* = 37) of farms included vehicle disinfection ford. From those with disinfection ford, Company 7 included nine farms (8.6% of 104 farms), Companies 1 included eight farms (11.4% of 70), and 3 included eight farms (11.6% of 69) each. Multiplier farms with disinfection fords were 47.6%, breeding herds 7.0%, nurseries 1.1%, finishers 0.5%, and farrow-to-finish farms 11.8%.

### Cluster analysis

**Figure 1:**
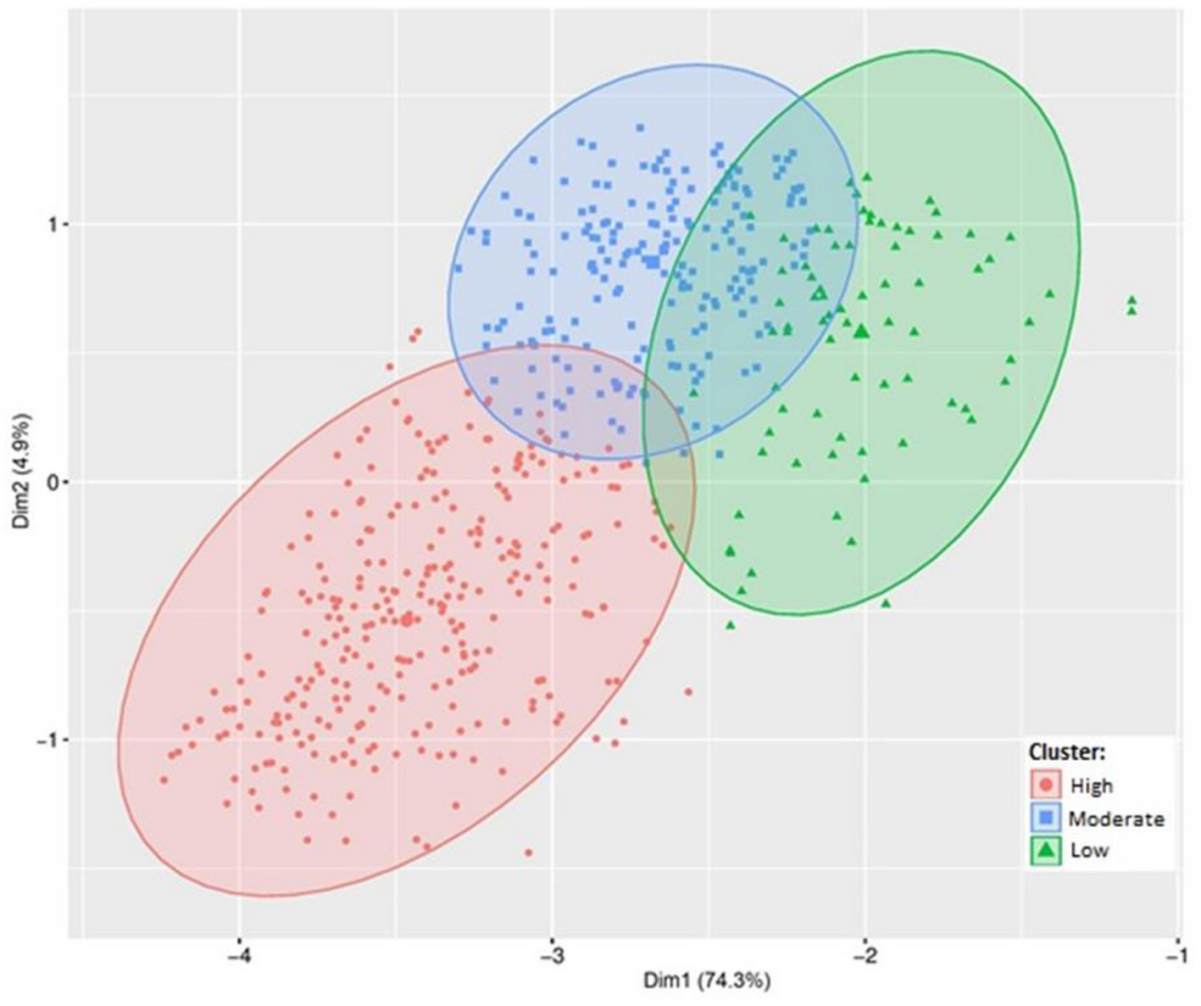
Cluster analysis of biosecurity compliance based on 21 biosecurity practice items required by the Federal Normative Instruction #44 (BRAZIL, 2017) in swine farms (*n* = 604) from the State of Rio Grande do Sul (Brazil). Custer 1 represent the “high compliant farms,” Cluster 2 “moderate compliant,” and Cluster 3 “low compliant.”

The cluster analysis showed that farms were classified as low, moderate, and high biosecurity compliant (*p*-value < 0.001; Figure 1; Table 2). Regardless the cluster classification, biosecurity compliance prevalently observed (~90%) were in terms of rodent controls, manure disposal, and feed formulation following guidelines from the Official Veterinary Service. For example, more than 99% of swine farms within each cluster formulate feed in accordance with the Official Veterinary Service's guidelines. In contrast, the practices of overall low compliance (<50%) were regarding bio-exclusion biosecurity, such as farm surroundings (presence of other species in swine farms, e.g., 27.1% swine farms in the high compliant cluster the staff personnel do not have contact with other livestock species) and barriers (disinfection chamber for material and ford for vehicles, lack of shower-in, e.g., only 10 and 4.0% swine farms in the high compliant cluster included disinfection chamber for materials and ford for vehicles). Overall, farms with high compliance (*n* = 303) performed 71% of the practices, moderate (*n* = 219) 47%, and the low (*n* = 82) 33%.

The frequency of farms and mean of biosecurity compliance by cluster within a swine commercial company is given in Table 3. The majority of farms (94%) from Company 5, followed by Company 7 (73%), were classified as high compliant. In contrast, most farms from Companies 2 (75%), 4 (75%), 8 (49%), and 9 (62%) were classified as moderate compliant.

**Table 3:** Frequency of farms and mean of biosecurity compliance (standard error) by cluster within a company.

**Company (*n* farms)**	**High compliant**		**Moderate compliant**		**Low compliant**	
	**(Cluster 1)**		**(Cluster 2)**		**(Cluster 3)**	
	**Freq. farms**	**Compliance mean** ^a^	**Freq. farms**	**Compliance mean** ^a^	**Freq. farms**	**Compliance mean** ^a^
1 (70)	56%	68% (0.10)	31%	47% (0.06)	13%	34% (0.04)
2 (79)	15%	67% (0.09)	75%	48% (0.06)	10%	32% (0.04)
3 (69)	58%	73% (0.11)	29%	47% (0.06)	14%	36% (0.06)
4 (4)	25%	90% (0.00)	75%	43% (0.04)	0%	–
5 (105)	94%	70% (0.10)	3%	48% (0.04)	3%	38% (0.07)
6 (30)	43%	59% (0.09)	37%	51% (0.04)	20%	39% (0.07)
7 (104)	73%	70% (0.11)	24%	44% (0.07)	3%	33% (0.00)
8 (73)	23%	68% (0.14)	49%	43% (0.06)	27%	34% (0.07)
9 (63)	5%	67% (0.04)	62%	44% (0.07)	33%	31% (0.07)
10 (7)	57%	81% (0.12)	14%	43% (0.00)	29%	33% (0.00)

a Compliance mean represents the average of biosecurity compliance by cluster within a Company. Biosecurity compliance is based on the requirements for Classical Swine Fever virus and Foot-month disease virus compartment certification, as described in chapters II e III from Federal Normative Instruction #44 (BRAZIL, 2017), see Table 1.

Custer 1 as “high compliant,” Cluster 2 as “moderate compliant,” and Cluster 3 as “low compliant.”

**Figure 2:**
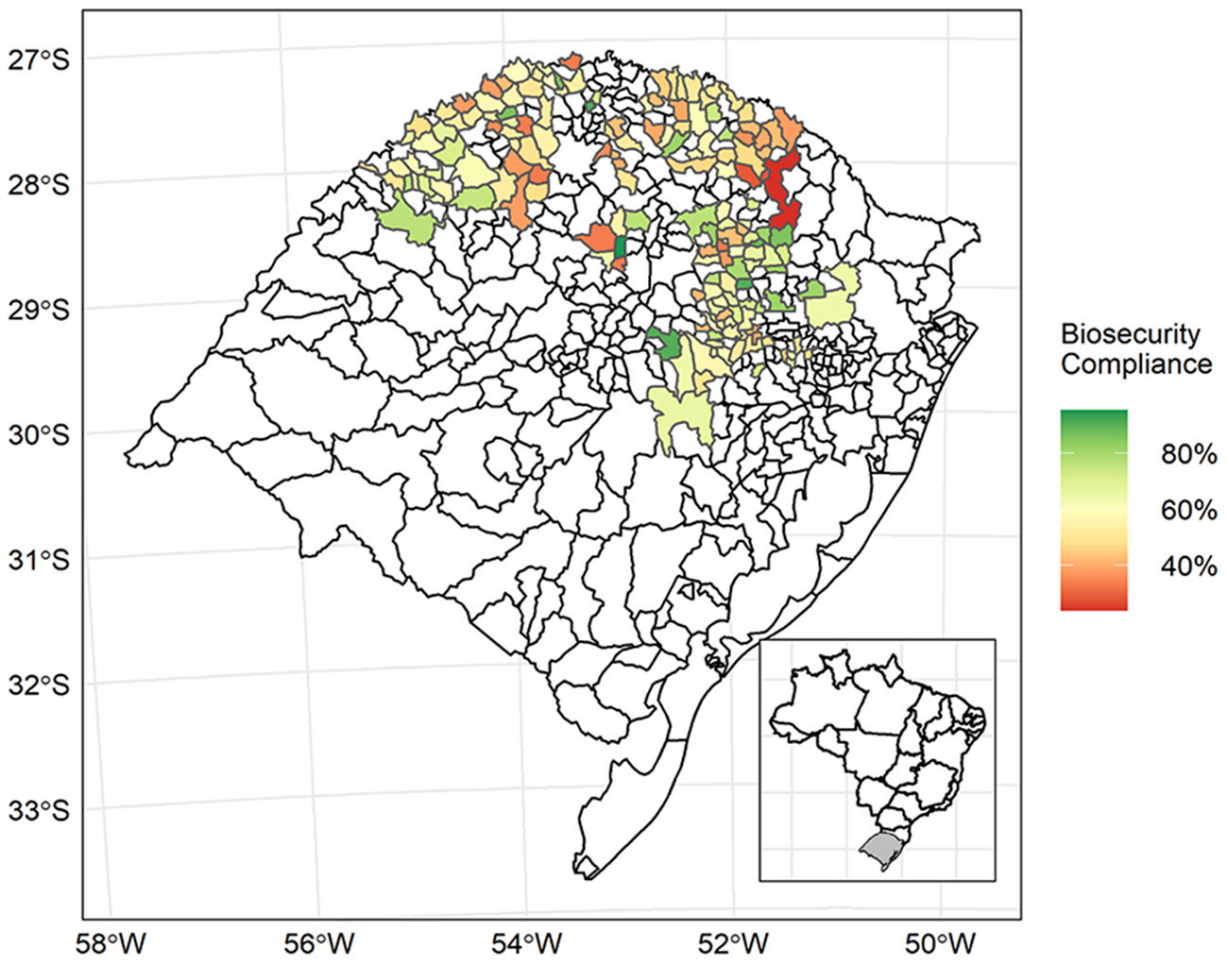
Frequency of biosecurity compliance in swine farms across sampled cities from the State of Rio Grande do Sul (Brazil).

**Figure 3:**
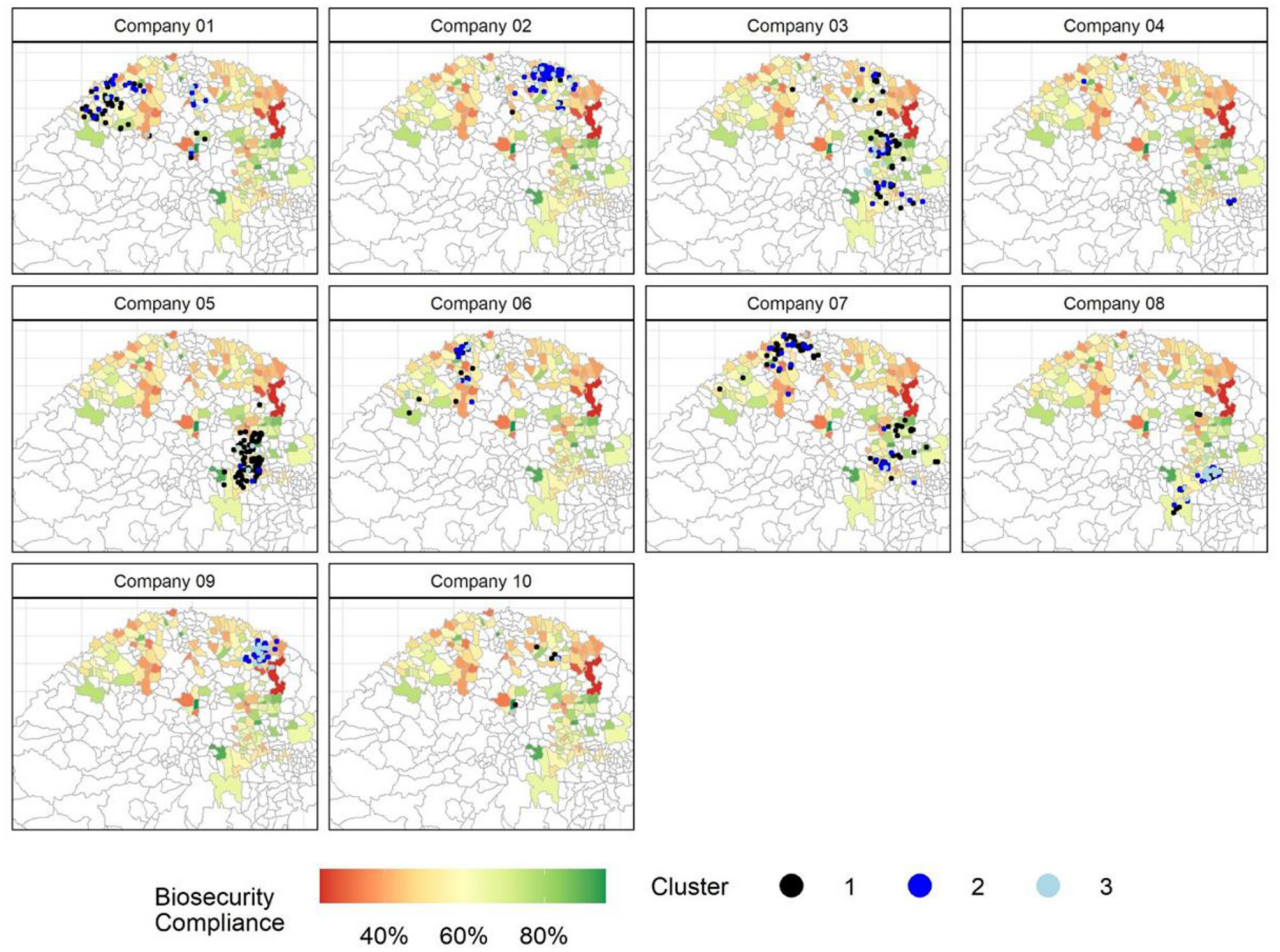
Distribution of swine farms (black dots) across sampled cities by companies and cluster from the State of Rio Grande do Sul (Brazil). Custer 1 as “high compliant,” Cluster 2 as “moderate compliant,” and Cluster 3 as “low compliant.”

The sampled swine farms included in this study were distributed in 163 cities of RS with an average of four farms sampled *per* city in RS Brazil (Figure 2). Figure 2 shows the frequency of biosecurity compliance by city of RS, and Figure 3 shows the location of sampled swine farms and their classification as either high, moderate, or low compliant. Overall, cities located in central-eastern and north-western regions from RS include farms with higher biosecurity compliance.

### Spatial regression modeling

Biosecurity compliance varied among swine companies and operational production types. The output from the final spatial logistic regression model is shown in Table 4. Company 5 resulted in the highest probability of biosecurity compliance (0.71, 95% CI 0.63, 0.78), followed by Company 7 (0.66, 95% CI 0.58, 0.74) and 4 (0.65, 95% CI 0.50, 0.77). In contrast, Companies 1, 2, 8, 9, and 10, all resulted in <60% probability of biosecurity compliance.

**Table 4:** Probability of niosecurity compliance and its relationship with swine company and operation production type, as estimated using a spatial logistic regression.

**Variable**	**Probability (95% CI)**
Intercept	–
**Swine company** ^a^	
1	0.59 (0.51, 0.68)^1, 2^
2	0.50 (0.41, 0.60)^1^
3	0.64 (0.56, 0.73)^2^
4	0.65 (0.50, 0.77)^2, 3^
5	0.71 (0.63, 0.78)^3^
6	0.59 (0.48, 0.68)^1, 2^
7	0.66 (0.58, 0.74)^2, 3^
8	0.49 (0.36, 0.59)^1^
9	0.42 (0.31, 0.55)^1^
10	0.60 (0.48, 0.70)^1, 2^
**Operational production types** ^a^	
Breeding	0.58 (0.50, 0.66)^2^
Multiplier	0.79 (0.72, 0.84)^3^
Farrow-to-finish	0.50 (0.40, 0.60)^1^
Nursery	0.55 (0.47, 0.63)^2^
Finishing	0.49 (0.41, 0.57)^1^

a Different superscripted number (1, 2, 3) indicates statistical differences (*p*-value ≤ 0.05) in the logit as estimated by the spatial logistic regression. Output was transformed to probabilities to facilitate interpretation.

Likewise, multiplier herds obtained a highest probability of biosecurity compliance (0.79, 95% CI 0.72, 0.84) compared to all other production operation types, e.g., breeding herd biosecurity compliance probability was equal to 0.58 (95% CI 0.50, 0.66), followed by nursery (0.55 95% CI 0.47, 0.63), farrow-to-finish (0.50, 95% CI 0.40, 0.60), and then finishing (0.49 95% CI 0.41, 0.57).

## Discussion

The technical background provided in Chapters II and III from the FNI #44 (BRAZIL, 2017) is in accordance with WOAH's compartmentalization guidelines and aims to isolate swine farms and their products within the compartment. In this sense, biosecurity and production management practices will work to mitigate the risks associated with sources of infection to surrounding farms within the compartment. Yet, not only the inclusion and execution of the proposed set of biosecurity practices will enable compartmentalization but also an ongoing inspection by the veterinarian authorities (3). Additionally, compartmentalization is expected to incentivize an increase in overall biosecurity compliance, promoting supplementary benefits. For instance, biosecurity compliance is known to be also essential to reach the overreaching goal of the swine industry to decrease antimicrobial usage (22). This study assessed biosecurity compliance to compartmentalization against CSF and FMD, as required by the FNI #44 (6) at the company and regional levels using data from a comprehensive biosecurity survey done in RS Brazil (13).

The overall biosecurity compliance varied among companies and operation production types, as reported and discussed previously (13). Different from previous study, a classification based on categories of biosecurity compliance was provided. Swine farms from high compliant group (Cluster 1) performed 71% of biosecurity practices, while moderate and low compliant groups performed <50%. As expected, due to the higher health standards, multiplier farms were all considered as high compliant group, except for multipliers from one company that were classified as moderate compliant. All other operation production types were classified as either high, moderate, and low compliant. This finding suggested that these latter operational production types (classified as moderate or low), e.g., breeding, nursery, and finishing, needed to work on improving biosecurity to be possibly incorporated in the compartment. As shown with poultry industries in the Netherlands (23), the transmission risk of highly pathogenic avian influenza increased when farms had different granting compartment statuses. Therefore, for a successful compartment, it is indicative that the entire system (from the breeder to the commercial production types) is granted equivalent biosecurity standards. High compliant farms (Cluster 1) were systematically compliant regarding biosecurity practices to truck transit, feed composition, and sanitation. Further, proper carcass and manure disposals were prevalently implemented in farms classified as either Cluster 1, 2, or 3. This is a positive aspect of swine farms in RS Brazil, given that not only CSF and FMD but also ASF viruses may survive in carcass and manure and potentially can cause an outbreak (24–26). In fact, many swine farms utilized composting as carcass disposal in this study (97%, data not shown), which was shown to be an effective strategy to mitigate soil contamination with FMD and other pathogens (27, 28).

However, the cluster analysis of biosecurity practices revealed a critical need for investment and overall improvement on bio-exclusion practices to lessen pathogen introduction. For instance, even in the higher compliant cluster, only 10.4 and 4.0% of farms included disinfection chamber for materials and disinfection ford for vehicles. Li et al. (29) described that ASF entered a large-scale Chinese commercial pig farm most likely through a contaminated vehicle used to market pigs that were poorly performing. Shower-in/shower-out coupled with using farm-specific clothes and boots for workers and visitors appeared to be implemented in ~74 and 57% of the swine farms classified into Cluster 1. To exemplify, Kim et al. (30) showed that the porcine epidemic diarrhea virus could be readily transmitted through contaminated personal protection equipment (PPE) to susceptible pigs. Another concerning performed practice in these farms is the contact of staff personnel with other species, e.g., at least 70% of the swine farms classified into the high compliant cluster reported physical contact with another livestock production (mainly poultry and dairy) in this study. Farmers and employees with contact with cattle during an FMD outbreak could carry the virus to the swine farm, causing an outbreak. Still the merely detection of FMD in a swine farm (backyard or commercial farms) can disrupt the international trade. Therefore, a comprehensive set of bio-exclusion security practices, such as physical barriers, is critical to preclude successfully the introduction of infectious pathogens in swine farms and guarantee the viability of the compartment.

The lack of bio-exclusion biosecurity practices exposes the swine commercial population to contacts with wild boars carrying infectious diseases. da Silva Andrade et al. and Varela et al. (31, 32) detected various pathogens from the porcine respiratory disease complex in wild boar, such as *Mycoplasma hyopneumoniae*, porcine circovirus 2, Influenza A virus, and porcine circovirus 3. These find can suggest that wild boar increase the pressure of infections with endemic disease in the commercial pig populations. While Silva et al. (33) detected antibodies against *Trichinella* spp. in wild boars, an important zoonotic disease. It was shown the risk posed by the wild boar population regarding the introduction of ASV in Europe (34). It is imperative that swine farms implement bio-exclusion biosecurity practices that impede the contact between commercial and wild pig populations.

At least 94% of swine farms from Company 5 were found in the high compliant cluster. This might facilitate the investment strategies because this company may focus on a specific set of biosecurity practices and lower number of farms. However, as shown in Table 3, Company 5 would need more investment in biosecurity practices related to bio-exclusion (barriers and farm surrounding) given their compliance were generally lower than sanitation, trucks, and feed. In contrast, other companies, such as 1, 3, and 7 included farms in all clusters, reflecting the need to invest in several types of practices, including mainly barrier (overall <35% compliance), farm surroundings, sanitation, and trucks. Yet, investment in biosecurity entails several factors that are related to individuality of farms, including cost, perception, facilities, and labor. The cost of bio-exclusion practices (shower-in/shower-out, disinfection fords, separation of clean from a dirty zone, etc.) can be higher and more likely limiting implementation, when compared to feed and trucks that can have lower cost (8, 35). Further, all these commercial companies would need to own at least one exclusive slaughterhouse plant, which is an important factor for compartment viability. Alternatively, different commercial swine companies but belonging to the same compartment could share slaughterhouse plants to maintain the compartment.

The spatial logistic model showed that biosecurity compliance to the FNI #44 was spatially correlated toward company and operation production type. In contrast, when analyzing biosecurity scores without the spatial component, other variables can affect biosecurity compliance, such as farm age, school level and experience in swine production of the farmer, and population size (13). Still, the spatial model provided reasonable results that can support the creation of a compartment. That is, one company (Company 5) resulted in the highest probability of biosecurity compliance (Table 4), which is the company that obtained the highest overall biosecurity compliance mean (Table 2), and highest portion of swine farms in the high compliant cluster (Table 3). Besides, Company 5 farms were mostly located in RS cities of higher biosecurity compliance (Figure 3).

As expected, biosecurity compliance of multipliers obtained the highest probability when compared to all other production types, and then they would be the first operation suitable to compartmentalization, meaning that they would not need initially larger investments. The challenge is to adequate the remaining operation production types given that the whole system should be part of the compartment. Alternatively, a company may strategize to create sub-compartments of higher compliant farms, which would secure the production from the compartment toward introduction of a foreign animal disease pathogen.

Nursery and finishing farms obtained lower probability of biosecurity compliance than breeding herds. In contrast, Silva et al. (13) described that nursery farms obtained higher biosecurity scores than breeding herds. This result can be explained by the use of different models, e.g., a spatial logistic vs. linear regression and a set of biosecurity practices, e.g., 21 vs. 35 biosecurity practices. However, it is recognized that nursery and finishing populations play a role on the maintenance and spread of disease (36). Therefore, regardless of methodology applied to evaluate biosecurity, it is imperative that southern Brazilian farms direct investments to these populations given that they include higher number of farms, higher number of movement events, and then receive animals from multiple sources, which sustainably contribute to the likelihood of disease epidemics.

## Conclusion

This study provided a methodology to assess the viability of compartment creation for swine-producing companies. Biosecurity compliance is key and required for compartmentalization for foreign swine diseases. In RS Brazil, one of 10 swine companies investigated would be more suitable to create a compartment for CSF and FMD with minimal investments in some biosecurity practices. This swine company showed higher indices of compliance in 21 biosecurity practice items required for compartmentalization in accordance with WOAH's guidelines (1, 2). Still, this study showed that improvements in biosecurity practices and compliance are urgently warranted in the majority of RS Brazil swine companies. Specific operational production types, such as the nursery and grow-finishing pig populations, need more attention regarding bio-exclusion biosecurity practices. Creating rapid response strategies to foreign animal diseases in free zones is imperative to secure livestock welfare, sustainability, and profitability.

## Data availability statement

The raw data supporting the conclusions of this article can be made available by the authors upon request.

## Author contributions

AS contributed to data collection, data analyses, and manuscript writing. KK contributed to data analyses and manuscript writing. AM contributed to the study design and data collection. LC and GS designed the study, coordinated data collection, analysis, and manuscript writing. All authors contributed to the article and approved the submitted version.
